# Planned Liver Stereotactic Body Radiotherapy for Residual Colorectal Cancer Liver Metastases After Surgery: A Single-Arm Retrospective Study

**DOI:** 10.3390/curroncol32060347

**Published:** 2025-06-12

**Authors:** Sixuan Li, Dezuo Dong, Xuan Zheng, Hongzhi Wang, Kun Wang, Baocai Xing, Weihu Wang

**Affiliations:** 1 Key Laboratory of Carcinogenesis and Translational Research (Ministry of Education), Department of Radiation Oncology, Peking University Cancer Hospital and Institute, Beijing 100142, China; sicons@pku.edu.cn (S.L.); dongdezuo@bjmu.edu.cn (D.D.); zhengxuan54321@163.com (X.Z.); wanghongzhi1988@126.com (H.W.); 2 Key Laboratory of Carcinogenesis and Translational Research (Ministry of Education), Department of Hepato-Biliary-Pancreatic Surgery I, Peking University Cancer Hospital and Institute, Beijing 100142, China; wang-kun@vip.sina.com

**Keywords:** colorectal cancer liver metastases, stereotactic body radiation therapy, curative-intent surgery, survival outcome

## Abstract

Given the promising outcomes of stereotactic body radiation therapy (SBRT) in treating colorectal cancer liver metastases (CRLMs), we proposed an innovative strategy combining surgery with planned liver SBRT for CRLMs. This retrospective study included patients who underwent curative-intent surgery combined with planned liver SBRT from July 2019 to October 2023. Planned liver SBRT was delivered to residual unresectable and unablatable lesions with maximum diameters of ≤5 cm. Outcomes included local failure (LF), intrahepatic recurrence-free survival (IHRFS), extrahepatic recurrence-free survival (EHRFS), progression-free survival (PFS), overall survival (OS), and radiation-related adverse events. A total of 69 patients were included. The 1-, and 2-year cumulative incidence rates of LF after SBRT were 7.7%, and 9.6%, respectively. The median PFS was 6.2 months, and the median OS was 45.8 months. Multivariate analysis identified RAS/BRAF mutations, extrahepatic metastases excluding lung involvement, and higher CEA as independent predictors of poorer OS. Intrahepatic recurrence was the predominant pattern of first disease progression after combination treatment. Acute grade 1–2 radiation-related adverse events occurred in 56.5% of patients, while grade 3 toxicities were reported in 4.3%. This approach offers favorable long-term outcomes, suggesting its potential to broaden the indications for curative-intent local treatments in CRLMs.

## 1. Introduction

Colorectal cancer (CRC) is the third most common malignancy, and the second leading cause of cancer-related deaths worldwide [1]. Liver remains the most frequent site of metastatic CRC. About 25% patients present concurrent colorectal cancer liver metastases (CRLMs) at the time of initial diagnosis, and up to 40–50% of patients will develop liver metastases during the course of the disease [2]. Surgical resection is a standard treatment for CRLM, however the majority of these metastases are considered as unresectable due to factors such as anatomical complexity, insufficient hepatic reserve, or other medical conditions [3].

For patients with unresectable lesions, a multidisciplinary team (MDT) plays a crucial role in conducting routine re-evaluation and integrating systemic therapies and local treatments tailored to individual cases. Local ablative therapies, including radiofrequency ablation (RFA), microwave ablation, and stereotactic body radiation therapy (SBRT), have been successfully implemented in patients with unresectable CRLMs [4,5]. RFA is regarded as a parenchymal-sparing treatment for hepatic malignancies, and is increasingly used combined with hepatectomy as a one-stage strategy for CRLMs [6]. To guarantee adequate tumor control, RFA is usually performed for lesions smaller than 2–3 cm and distant from vulnerable structures [4]. Local tumor recurrence rate after RFA ranges between 8 and 25% [7,8,9], and combined ablation and resection (CARe) has been suggested to offer satisfactory oncological outcomes for patients with multifocal disease [10,11]. Meanwhile, SBRT offers an alternative, non-invasive approach through precisely targeted delivery of highly conformal radiation in one or a few fractions to tumors. Liver SBRT is utilized due to fewer limitations of tumor size and vascular vicinity, and has achieved comparable local control rates to other ablative techniques, making it an appealing option for patients who are not candidates for surgery or RFA [12,13]. Notably, recent studies have demonstrated that SBRT is a safe and effective local treatment for CRLMs, with 1-year local control rates exceeding 90% [14,15,16].

Recent advancements in systemic therapies, including targeted agents and immunotherapies, have further improved outcomes for patients with CRLMs. Although effective systemic therapies can eliminate micro-metastases, they are insufficient to achieve complete tumor eradication [4], underscoring the critical role of local treatments to control disease progression and improve long-term survival. Consequently, there is an urgent demand for innovative strategies to address unresectable and unablatable CRLMs. Given the promising outcomes of SBRT, we hypothesized that integrating planned liver SBRT into the treatment paradigm could be a novel strategy for patients with unresectable and unablatable lesions. Hopefully, the combination treatment would expand the indications for curative-intent local treatments, and improve prognosis for patients with unresectable and unablatable CRLMs who currently have limited treatment options.

We hypothesized that combining surgery with planned liver SBRT offers a safe and effective local treatment strategy for CRLMs, capable of achieving durable local control and potentially improving overall survival. Therefore, this study aimed to evaluate the safety, short-term, and long-term outcomes of planned liver SBRT for residual CRLMs following surgery.

## 2. Materials and Methods

### 2.1. Patient Selection

**Figure 1:**
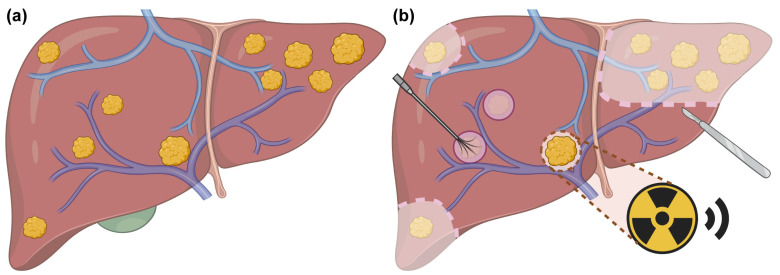
Schematic illustration of surgery combined with planned liver SBRT for CRLMs. (**a**) Liver with multiple metastatic lesions before surgery and planned liver SBRT. (**b**) Sectionectomy is performed for multiple lesions located in the left lobe. Wedge resection is applied for superficial lesions in the right lobe. Intraoperative ablation is preferred for small lesions (diameter < 2 cm) that are deeply located and sufficiently distant from adjacent hepatic vein and bile duct. The lesion located in the first porta hepatis, deemed as unresectable and unablatable, is selected for planned liver SBRT. Created with biorender.com.

This study included consecutive CRLM patients who underwent surgery combined with planned liver SBRT at Peking University Cancer Hospital. A preliminary treatment strategy was developed at the initial evaluation. Patients were re-evaluated every 2 cycles of pre-treatment systemic therapies, and those with evidence of disease progression were ineligible for subsequent local treatments. Prior to surgery, all patients underwent an MDT consultation to determine lesion-specific local treatment plans. Based on the findings from the latest preoperative MRI, the MDT collectively identified lesions unsuitable for resection or ablation, and simultaneously assessed the feasibility of planned liver SBRT for these cases. Following hepatic surgery, the radiation oncologist would reassess the intraoperative findings and the patient’s recovery status to promptly deliver planned liver SBRT to residual lesions. Additionally, the predicted future liver remnant volume after treatment was required to exceed 30% of the total liver volume. Surgical approaches included hepatic resection alone and CARe. Radiotherapy was scheduled promptly after the normalization of inflammatory and liver function markers, complete wound healing, drain removal, and the patient’s return to normal daily activities. Planned liver SBRT was typically initiated within 5–12 weeks after surgery, with concurrent systemic therapy permitted. Lesions selected for planned liver SBRT were identified as unresectable and unablatable during the pre-operative MDT meeting or intraoperatively, with maximum diameters ≤5 cm (Figure 1). Unresectable and unablatable CRLMs were defined as tumors located near the first or the second porta hepatis, involving or adjacent to the following: (1) main portal vein or its major branches; (2) major hepatic veins; (3) major intrahepatic bile ducts. In such cases, complete resection would lead to insufficient volume of functional liver parenchyma, while complete ablation would pose a risk of damaging vital structures.

Patients were excluded if they met the following criteria: (1) develop disease progression after pre-treatment systemic therapies; (2) lack of pre-treatment MRI or post-treatment radiological evaluation; (3) history of other malignancies. Presence of limited, stable extrahepatic distant metastasis was not necessarily an exclusion criterion. This study was approved by the Ethics Committee of Peking University Cancer Hospital (2024YJZ146). Written informed consent was obtained from all patients.

### 2.2. Treatment Procedures

#### 2.2.1. Systemic Treatment

For most patients, pre-operative first-line treatment comprised an irinotecan or oxaliplatin-based doublet chemotherapy regimen, with or without targeted agents. Doublet chemotherapy plus cetuximab was the preferred option for left-sided, RAS and BRAF wild-type tumors, whereas for right-sided or RAS-mutant tumors, the addition of bevacizumab was generally recommended. Selected patients with good performance status received triplet chemotherapy combined with bevacizumab to enhance tumor response. Patients who did not respond to first-line therapy were not necessarily excluded from local interventions if disease control was achieved through second-line conversion therapy [5]. The decision to administer post-operative systemic therapy was discussed at MDT meetings based on Fong’s clinical risk score and Sasaki’s tumor burden score [17,18]. Intra-arterial therapies would be considered as alternative options when patients were intolerant to intravenous therapy or tumor burden reduction was inadequate. Tumor response to systemic treatment was evaluated every 2 cycles according to RECIST v1.1.

#### 2.2.2. Surgical Interventions

Surgery was performed 4–6 weeks after the last cycle of pre-operative systemic therapy. The surgical technique was previously described in [6,19]. In brief, resection strategies included hemihepatectomy, sectionectomy, segmental resection, and wedge resection. CARe was conducted when tumors were unable to be completely removed by single hepatectomy or deeply located in the remnant liver. Ablated lesions were required to have a diameter of <2 cm. Intraoperative RFA was conducted using probes inserted directly into the tumor under ultrasound guidance, with repeated ablations performed as necessary. Extrahepatic diseases, such as lymph nodes, were synchronously resected when complete clearance was feasible.

#### 2.2.3. SBRT Techniques

The detailed procedures were described in our previous study [20]. All patients underwent respiratory training before simulation to ensure consistent breathing patterns. Abdominal compression was used in tolerable patients to minimize respiratory motion. For treatment planning, enhanced CT and MRI scans (3–5 mm layer thickness) were obtained, with two-phase T2-weighted images acquired at the end of inspiration and expiration. Image registration between simulation CT and MRI was performed using the Eclipse treatment planning system (Varian, Palo Alto, CA, USA). The gross tumor volume (GTV) was delineated on fused CT-MRI images with reference to preoperative diagnostic images. The internal target volume (ITV) encompassed the sum of GTVs over the respiratory cycle. And the planning target volume (PTV) was created by adding a 5 mm margin to the ITV. SBRT was defined as delivering 5 Gy per fraction or greater. The prescribed dose was designed to achieve 95% PTV coverage. Treatment was delivered with 6-MV X-rays linear accelerator.

### 2.3. Endpoint Assessment

Efficacy endpoints included local failure (LF), intrahepatic recurrence-free survival (IHRFS), extrahepatic recurrence-free survival (EHRFS), progression-free survival (PFS), and overall survival (OS). LF was defined as radiologically proven failure of the treated lesions, including recurrence in or at the margin of the RFA site or resection margin, as well as lesion developing within the PTV after planned liver SBRT. IHRFS was defined as time from the date of combination treatment to recurrence within the liver outside of the treated lesions. EHRFS was defined as time from the date of combination treatment until disease progression in other metastatic sites outside of liver. PFS was defined as time from the date of combination treatment until disease progression at any location, appearance of new lesions, or death, whichever occurred first. OS was defined as time from the date of combination treatment to death or last follow-up.

Safety endpoints included toxicities. Radiation-related toxicities were evaluated using the Common Toxicity Criteria Adverse Events version (CTCAE) v4.0. Acute toxicities were defined as adverse events occurring within 3 months post-SBRT, while late toxicities were defined as those occurring after 3 months.

### 2.4. Statistical Analysis

Continuous variables were summarized as median (range), and categorical variables were described by percentages. The cumulative incidence function was used to estimate LF after SBRT, with death as a competing risk. IHRFS, EHRFS, PFS, and OS were evaluated using the Kaplan–Meier analysis. Variables with significant associations in univariate analyses were further analyzed using multivariate Cox regression models, with results presented as hazard ratios (HRs) and 95% confidence intervals (CIs). Fisher’s exact test was used to compare categorical variables. Statistical analyses were performed using R (version 4.1.0, R Foundation for Statistical Computing), with a two-sided *p*-value < 0.05 considered statistically significant.

## 3. Results

### 3.1. Patient and Treatment Characteristics

From July 2019 to October 2023, a total of 69 patients were included in the analysis. The clinicopathological characteristics of these patients are summarized in Table 1. Most patients (88.4%) presented with bilobar liver metastases, with a median number of 11 lesions. Of the 69 patients, 79.7% had no extrahepatic metastases, while among patients with extrahepatic metastases, lung was the most frequent site.

Table 2 summarizes the primary treatment characteristics. Pre-treatment systemic therapy was administered to 71.0% of patients as first-line therapy and to 29.0% as second-line therapy. Additionally, 85.5% of patients received post-treatment systemic therapy. CARe was performed in 43 patients (62.3%), particularly in those with a greater number of lesions. The median interval between surgery and planned liver SBRT was 46 days (range, 22–107 days). A total of 123 lesions were treated by SBRT, with a median of one lesion per patient (range, 1–5 lesions). Total doses ranging from 42.0 to 65.0 Gy were delivered to patients in 5 to 12 fractions without interruption. When converted into the biologically effective dose (BED_10_), the median dose was 100.0 Gy (range, 71.4–132.0 Gy). A detailed summary of the anatomical locations of SBRT-treated lesions is provided in Table S1.

### 3.2. Oncological Outcomes

LF was observed in 13 patients (18.8%) at the last follow-up. Among these, seven patients (10.1%) experienced LF following surgery, while six patients (8.8%) developed LF after SBRT. The cumulative incidence rates of LF after SBRT per patient, accounting for death as a competing risk, were 7.7% at 1 year and 9.6% at 2 years. And the 1-, and 2-year cumulative incidence rates of LF after SBRT per lesion were 6.0%, and 7.2%, respectively.

**Figure 2:**
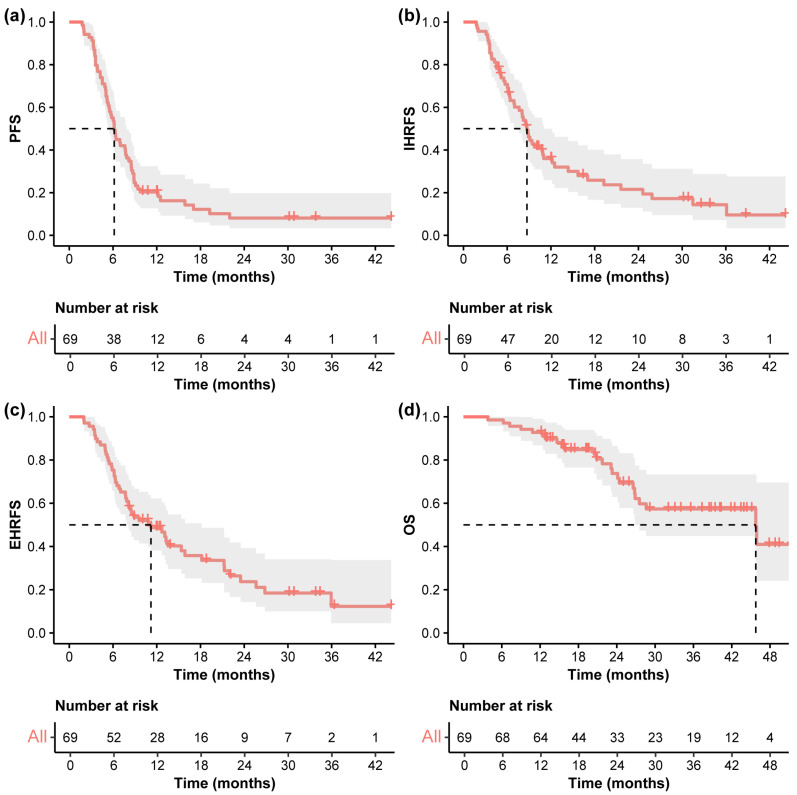
Kaplan–Meier curves. Kaplan–Meier curves of (**a**) PFS, (**b**) IHRFS, (**c**) EHRFS, and (**d**) OS in the entire cohort. The dashed line indicates the median survival time. Abbreviations: PFS, progression-free survival; IHRFS, intrahepatic recurrence-free survival; EHRFS, extrahepatic recurrence-free survival; OS, overall survival.

The median follow-up was 35.6 months (95% CI 25.9–41.9). As shown in Figure 2, the median PFS was 6.2 months (95% CI 5.5–7.9), and the PFS was 20.1%, and 8.0% at 1, and 2 years, respectively. The median IHRFS was 8.7 months (95% CI 7.0–12.2), with the 1-, and 2-year IHRFS was 36.0%, and 21.5%, respectively. The median EHRFS was 11.2 months (95% CI 8.1–18.2), and the EHRFS was 48.7%, and 23.7% at 1, and 2 years, respectively. The median OS was 45.8 months (95% CI 26.9-NA), and the OS at 1, 2, and 3 years was 92.8%, 73.8%, and 57.4%, respectively.

### 3.3. Pattern of Progression

The patterns of first disease progression were consistent between the entire cohort and the subgroup of patients with liver-only metastases at baseline (Table S2). Among the entire cohort, intrahepatic recurrence was the predominant pattern of first progression, observed in 31 patients (44.9%). Similarly, in the subgroup of patients with liver-only metastases, intrahepatic recurrence remained the predominant pattern, occurring in 31 patients (49.1%). For patients who developed extrahepatic progression, lung was the most frequent site (18.8%), followed by lymph nodes (7.2%) and peritoneum (5.8%).

### 3.4. Prognostic Analysis for Survival Outcomes

Cox regression analysis identified several prognostic factors associated with OS (Table 3). In the univariate analysis, significant predictors included RAS/BRAF mutation (*p* = 0.004), extrahepatic metastases excluding lung involvement (*p* < 0.001), and higher levels of CEA (*p* = 0.010). Multivariate analysis confirmed these factors as independent predictors of worse OS.

Similarly to the findings for OS, RAS/BRAF mutation (*p* = 0.001), and extrahepatic metastases excluding lung involvement (*p* = 0.009) were independently associated with poorer EHRFS. Conversely, the number of liver metastases was identified as the only independent prognostic factor for IHRFS (Table S3).

### 3.5. Toxicity

Grade 1–2 radiation-related acute adverse events occurred in 39 patients (56.5%), with grade 3 severe toxicities reported in only three patients (4.3%), all of which were hematologic toxicities (Table 4). No additional late toxicities were noted during the follow-up period.

## 4. Discussion

Current studies on local treatments for CRLMs primarily concentrate on comparing the indications and effectiveness of various techniques. In contrast, we propose a novel strategy combining planned liver SBRT with surgery, rather than utilizing SBRT alone as an alternative treatment. The present study demonstrates that planned liver SBRT is safe and effective, thereby expanding the eligibility for curative treatments to encompass unresectable and unablatable CRLMs.

SBRT for liver metastases has demonstrated promising results, with local control rates ranging from 70 to 100% at 1 year and 60–90% at 2 years [16,21,22,23]. A prior study conducted at our institution showed that by using 4D-MRI simulation and respiratory control techniques, the local control rate at 1 year for unresectable CRLMs treated with SBRT alone was 97.3% [20]. Consistently, in this study, the 1-, and 2-year cumulative incidence rates of LF after SBRT per patient were 7.7%, and 9.6%. Despite the high local control rate, a large proportion of patients developed out-of-field intrahepatic recurrence and extrahepatic disease progression. Intrahepatic recurrence is a common pattern of progression following surgical resection for CRLMs. A meta-analysis of 24 studies involving 12,705 patients found that 30.2% of CRLM patients experienced early recurrence after liver resection [24]. And previous studies focusing on surgical resection and CARe in patients with multiple lesions reported the median IHRFS ranging from 8 to 30 months and median PFS from 7 to 22 months (Table S4) [6,19,25,26,27,28]. The novel treatment strategy in our study showed a median IHRFS of 8.7 months (95% CI 7.0–12.2) and a median PFS of 6.2 months (95% CI 5.5–7.9), slightly inferior to those reported for surgical treatment. Given that Cox regression identified the number of lesions as the only factor significantly associated with poorer IHRFS, we hypothesized that intrahepatic recurrence may be attributed to the greater tumor burden in our cohort. In contrast to most studies, where the median number of lesions is fewer than five, 50% of patients in our cohort had more than ten metastases. Notably, a previous study at our center found that among 67 patients with multiple CRLMs (median number of 10 lesions per patient) who received CARe, the median IHRFS and PFS were 8 months and 7 months, respectively, which are consistent with the results of this study. We also compared our results with key trials evaluating first-line chemotherapy for initially unresectable CRLMs (Table S5) [29,30,31,32,33]. These studies reported median PFS ranging from 9 to 12 months, which appears longer than the 6.2 months observed in our cohort. However, this discrepancy should be interpreted with caution for several reasons. First, PFS in these clinical trials was typically calculated from the initiation of chemotherapy, whereas we defined PFS from the date of surgery. Second, in the CAIRO5 study [33], the maximum number of liver metastases was 24, and over 90% of patients had unilobar disease. In contrast, patients in our cohort had higher tumor burden, with 17% of patients exceeding 24 metastases and 88.4% presenting with bilobar involvement. As previously discussed, higher lesion number is associated with shorter PFS. Moreover, the CELIM and CAIRO5 studies reported that over 30% of patients were successfully down-staged to resectable status after systemic therapy, and subsequently received curative-intent surgery [29,33]. Previous studies have shown that patients achieving R0 resection show significantly better survival outcomes [29], suggesting that the PFS benefit observed in these trials may be attributable to surgical resection rather than chemotherapy alone. Despite this, our cohort achieved a median OS of 45.8 months, comparable to outcomes of CRLMs receiving hepatectomy or CARe (Table S4) [6,26,27,28,34,35,36]. Although achieving long-term disease-free status is crucial, OS remains the most optimal endpoint for metastatic CRC. According to the largest comparative analysis of survival outcomes after curative-intent hepatectomy for CRLMs, there was minimal correlation between PFS and OS [37]. The investigators concluded that OS was primarily influenced by the pattern of progression and subsequent therapies, rather than the interval before disease progression. This may partly explain why, despite shorter PFS, our approach still resulted in significant OS benefits. Moreover, when compared with systemic therapies, our cohort’s median OS exceeded the 22 to 40 months reported in previous trials (Table S5) [29,30,31,32,33]. This further highlights the importance of curative-intent local treatments in the management of CRLMs.

To enhance this treatment modality, we identified several factors associated with OS. Consistent with many predictive models of CRLMs, patients with RAS/BRAF mutation showed significantly worse survival (*p* = 0.010). Notably, our findings indicated that the location of metastases, particularly metastases outside the liver and lungs, had a greater impact on OS than the number of lesions. Although several studies have elucidated the number of metastases as a prognostic factor for long-term survival after ablative treatments, typically stratifying patients with no more than 5–10 lesions as the best candidates for local therapy [38], many experts argue that the feasibility of safely delivering metastases-directed local treatment determines the maximum number of lesions [39]. Similarly, our findings emphasized the importance for curative-intent local treatments in metastatic CRC, particularly when metastases are confined to the liver and lungs. At present, patients with tumors located near major hepatic vessels, bile ducts are often excluded from curative-intent local treatments due to the risk of severe liver failure or incomplete treatment. In our cohort, planned liver SBRT allowed precise targeting of lesions near critical structures without compromising their safety, indicating that this approach can effectively treat a higher number of metastases and lesions in more anatomically challenging locations compared to conventional local treatments.

Meanwhile, our study indicated that surgery combined with planned liver SBRT was safe and tolerable. Grade 3 radiation-related adverse events were observed in only three patients (4.3%), all of whom experienced hematologic toxicities and underwent high-intensity post-operative chemotherapy. Notably, none of our patients developed signs of liver failure or jaundice. These results are consistent with previously reported low toxicity rates after liver SBRT. In a systematic review including 18 studies with a total of 656 patients, the pooled incidence of severe liver toxicity was reported to be 8.7%, with rare cases of liver failure (0.6%) [13]. The low incidence of toxicities observed in our study may be attributed to the fact that (1) CARe preserves sufficient liver parenchyma; (2) introduction of MRI improves the accuracy of delineation, and adequately spares the normal tissues; and (3) abdominal compression during SBRT reduces the irradiation volume [20].

The study has several limitations. First, this single-arm, retrospective study included only 69 patients, which limits its statistical power and generalizability. As no formal sample-size calculation was performed prior to the study, the results should be interpreted with caution. Larger, prospective studies with predefined power analyses are needed to validate our findings and confirm their applicability in broader clinical settings. Second, the absence of a control group limits direct comparison with alternative strategies, including other local treatment modalities or systemic chemotherapy. Multi-arm or propensity-matched studies are necessary to evaluate the relative efficacy of different treatment strategies for CRLMs. Additionally, future research should explore incorporating novel biomarkers and advanced imaging techniques, such as multi-parametric MRI, to better identify patients likely to benefit from planned liver SBRT. Longer follow-up is also needed to provide more accurate data on long-term oncological outcomes.

## 5. Conclusions

In summary, the novel strategy that combines planned liver SBRT with surgery is safe and effective for patients with unresectable and unablatable CRLMs. Although a large proportion of patients in our cohort developed intrahepatic recurrence, the overall survival in this study showed promising results, suggesting the combination treatment would expand the indications for curative-intent local treatments in CRLMs.

## Figures and Tables

**Table 1:** Patient characteristics.

Patient Characteristic	Patients, No. (%)
Age, median (range)	58 (30–72)
Gender	
Male	47 (68.1%)
Female	22 (31.9%)
ECOG PS	
0	59 (85.5%)
1	10 (14.5%)
Primary tumor location	
Right colon	7 (10.1%)
Left colon	41 (59.4%)
Rectum	21 (30.4%)
T stage	
2	2 (2.9%)
3	52 (75.4%)
4	15 (21.7%)
N stage	
0	15 (21.7%)
1	39 (56.5%)
2	15 (21.7%)
Gene testing	
Wild-type	42 (60.9%)
RAS mutation	24 (34.8%)
BRAF mutation	4 (5.8%)
Timing of liver metastases	
Synchronous metastases	60 (87.0%)
Metachronous metastases	9 (13.0%)
Number of CRLMs per patient, median (range)	11 (2–58)
Distribution of liver metastases	
Unilobar metastases	8 (11.6%)
Bilobar metastases	61 (88.4%)
Location of extrahepatic metastatic sites	
None	55 (79.7%)
Lung	8 (11.6%)
Others	6 (8.7%)

Abbreviations: ECOG PS, Eastern Cooperative Oncology Group Performance Status; CRLMs, colorectal cancer liver metastases.

**Table 2:** Treatment characteristics.

Treatment Parameters	Patients/Lesions, No. (%)
Pre-treatment systemic therapy	
First-line	49 (71.0%)
Second-line	20 (29.0%)
Best response of pre-treatment systemic therapy	
Partial response	44 (63.8%)
Stable disease	25 (36.2%)
Post-treatment systemic therapy	
No	10 (14.5%)
Yes	59 (85.5%)
Type of surgical approach	
CARe	43 (62.3%)
Hepatectomy	26 (37.7%)
Interval between surgery and SBRT, median (range)	6.6 weeks (3.1–15.3)
≤5 weeks	10 (14.5%)
5–10 weeks	50 (72.5%)
>10 weeks	9 (13.0%)
Number of CRLMs treated by different surgical approaches per patient	
CARe, median (range)	13 (3–57)
Hepatectomy, median (range)	5 (1–10)
Number of CRLMs treated by SBRT per patient, median (range)	1 (1–5)
Size of CRLMs treated by SBRT, median (range)	14 mm (5–42)
<10 mm	22 (17.9%)
10–19 mm	60 (48.8%)
20–29 mm	27 (22.0%)
≥30 mm	14 (11.4%)
ITV, median (range)	13.1 cm^3^ (0.7–90.1)
<5.0 cm^3^	20 (16.3%)
5.0–24.9 cm^3^	71 (57.7%)
25.0–49.9 cm^3^	24 (19.5%)
≥50.0 cm^3^	8 (6.5%)
PTV, median (range)	38.5 cm^3^ (4.0–173.4)
<20.0 cm^3^	16 (13.0%)
20.0–59.9 cm^3^	74 (60.2%)
60.0–99.9 cm^3^	23 (18.7%)
≥100.0 cm^3^	10 (8.1%)
BED_10_ per lesion, median (range)	100.0 Gy (71.4–132.0)
≥100.0 Gy	70 (56.9%)
<100.0 Gy	53 (43.1%)

Abbreviations: CARe, combined ablation and resection; CRLMs, colorectal cancer liver metastases; SBRT, stereotactic body radiation therapy; ITV, internal target volume; PTV, planning target volume; BED, biologically effective dose.

**Table 3:** Univariate and multivariate analyses used to determine predictors of overall survival.

Variables	Univariate	Multivariate	
	*p*-Value	HR (95% CI)	*p*-Value
Gender (male vs. female)	0.690		
Age (years)	0.856		
Primary tumor			
Location (left-sided vs. right-sided)	0.589		
Stage (N1–2 vs. N0)	0.272		
RAS/BRAF status (mutation vs. wild-type)	0.004	4.49 (1.69–11.95)	0.003
Liver metastases			
Synchronous vs. metachronous	0.498		
Distribution (bilobar vs. unilobar)	0.158		
Number of lesions	0.218		
Maximum lesion size at diagnosis (mm)	0.590		
Extrahepatic metastases			
None	Ref	Ref	Ref
Lung	0.789	0.98 (0.21–4.48)	0.978
Others	<0.001	15.73 (4.06–60.96)	<0.001
CEA (ng/mL)	0.010	1.00 (1.00–1.01)	0.041
Pre-treatment therapy			
First-line vs. second-line	0.509		
Best response (SD vs. PR)	0.128		

Abbreviations: HR, hazard ratio; CI, confidence interval; CEA, carcinoembryonic antigen; SD, stable disease; PR, partial response.

**Table 4:** Radiation-related acute adverse events.

	Grade 1 No. (%)	Grade 2 No. (%)	Grade 3 No. (%)
Leukopenia	17 (24.6%)	15 (21.7%)	2 (2.9%)
Neutropenia	15 (21.7%)	13 (18.8%)	0 (0.0%)
Thrombocytopenia	10 (14.5%)	4 (5.8%)	1 (1.4%)
Anemia	13 (18.8%)	1 (1.4%)	0 (0.0%)
Elevated ALT/AST	10 (14.5%)	1 (1.4%)	0 (0.0%)
Hyperbilirubinemia	7 (10.1%)	0 (0.0%)	0 (0.0%)
Abdominal pain	0 (0.0%)	1 (1.4%)	0 (0.0%)
Diarrhea	3 (4.3%)	0 (0.0%)	0 (0.0%)
Nausea	6 (8.7%)	0 (0.0%)	0 (0.0%)
Fatigue	4 (5.8%)	0 (0.0%)	0 (0.0%)

Abbreviations: ALT, alanine aminotransferase, AST, aspartate aminotransferase.

## Data Availability

The original contributions presented in this study are included in the article/Supplementary Materials. Further inquiries can be directed to the corresponding authors.

## Supplementary Materials

The following supporting information can be downloaded at: https://www.mdpi.com/article/10.3390/curroncol32060347/s1, Table S1: Anatomical locations of lesions treated by SBRT; Table S2: Patterns of first disease progression; Table S3: Univariate and multivariate analyses to determine predictors of intrahepatic recurrence-free survival and extrahepatic recurrence-free survival; Table S4: Studies of hepatectomy alone or combined ablation and resection for CRLMs in the recent 5 years; Table S5: Key clinical trials of first-line chemotherapy for initially unresectable CRLMs.
