# Supplementary material for: Planned Liver Stereotactic Body Radiotherapy for Residual Colorectal Cancer Liver Metastases After Surgery: A Single-Arm Retrospective Study
Source: Curr Oncol. 2025 Jun 12;32(6):347. doi: 10.3390/curroncol32060347 (PMC12191733; doi:10.3390/curroncol32060347)
Supplement: Supplementary file 1 [file curroncol-32-00347-s001.zip › curroncol-3606167-supplementary.pdf]

Table S1. Anatomical locations of lesions treated by SBRT.

No.	Lesion location [a]	No.	Lesion location [a]	No.	Lesion location [a]
1	S4a (MHV), S8 (MHV & PV)	24	S7 (RHV)	47	S5 (RPV)
2	S1 (IVC & PV)	25	S5 (RHV)	48	S1 (IVC), S7 (RHV)
3	S2 (the root of LHV)	26	S4 (MHV), S7 (RHV), S1 (IVC), S5/8 (RHD)	49	S4 (MHV)
4	S2 (the root of LHV), S7 (RHV & RHD)	27	S4 (the second porta hepatis)	50	S1 (IVC)
5	S8 (MHV), S8 (PV)	28	S1 (IVC)	51	S1 (IVC)
6	S1 (IVC & PV)	29	S5 (RPV)	52	S5/8 (RHD)
7	S1 (IVC & PV)	30	S4/8 (the second porta hepatis), S5 (RPV)	53	S4 (MHV), S7 (RHV), S5 (RHV), S8 (RHV)
8	S8 (MHV)	31	S8 (the root of RHV)	54	S2 (the root of LHV), S1 (IVC & PV)
9	S5 (RHV), S6 (RPV)	32	S8 (MHV), S8 (the root of RHV)	55	S4/8 (MHV)
10	S5 (RHV), S1 (IVC & PV)	33	S7 (the root of RHV)	56	S1 (IVC & PV)
11	S1 (IVC & PV)	34	S1 (IVC)	57	S5 (RHV)
12	S7 (RHV)	35	S4/8 (the second porta hepatis)	58	S4/8 (MHV)
13	S7 (IVC), S4/8 (the second porta hepatis)	36	S4/8 (the second porta hepatis), S1 (IVC)	59	S1 (IVC)
14	S2 (the root of LHV), S1 (IVC & PV)	37	S4 (MHV)	60	S7 (RHV), S8 (MHV), S5/8 (MHV)
15	S2/3 (LPV)	38	S4 (MHV)	61	S1 (IVC), S8 (MHV)
16	S5 (RHV & RHD)	39	S6 (RPV)	62	S1 (IVC), S8 (MHV & PV)
17	S1 (IVC)	40	S5 (RHV)	63	S7/8 (RHV), S8 (MHV)
18	S3 (LPV)	41	S5/8 (MHV)	64	S4 (MHV)
19	S7 (RHV)	42	S8 (MHV)	65	S1 (IVC)
20	S4/8 (the second porta hepatis)	43	S4a (LPV), S8 (MHV)	66	S8 (MHV)
21	S8 (MHV)	44	S1 (IVC & PV)	67	S5 (RPV)
22	S1 (IVC)	45	S4 (LPV & LHD), S6 (RPV & posterior branch of RHD)	68	S4/8 (the second porta hepatis)
23	S1 (IVC)	46	S6 (RPV), S4 (MHV)	69	S2 (the root of LHV), S8 (RHV)

[a] Couinaud segment (adjacent/involved structures). Abbreviations: IVC, inferior vena cava; PV, portal vein; RPV, right portal vein; LPV, left portal vein; MHV, middle hepatic vein; RHV, right hepatic vein; LHV, left hepatic vein; RHD, right hepatic duct; LHD, left hepatic duct.

Table S2. Patterns of first disease progression.

	Entire cohort (N = 69)	Liver-only (N = 55)	P value *
Disease free	8 (11.6%)	7 (12.7%)	1.000
Intrahepatic recurrence	31 (44.9%)	27 (49.1%)	0.718
New metastases	28 (40.6%)	24 (43.6%)	0.855
LF	2 (2.9%)	2 (3.6%)	1.000
LF + new metastases	1 (1.4%)	1 (1.8%)	1.000
Extrahepatic disease progression	26 (37.7%)	18 (32.7%)	0.578
Lung	13 (18.8%)	9 (16.4%)	0.815
Lymph nodes	5 (7.2%)	4 (7.3%)	1.000
Peritoneum	4 (5.8%)	2 (3.6%)	0.692
Others	4 (5.8%)	3 (5.5%)	1.000
Intrahepatic recurrence + extrahepatic disease progression	4 (5.8%)	3 (5.5%)	1.000

* Comparisons were made by the Fisher's exact test. Abbreviations: LF, local failure.

Table S3. Univariate and multivariate analyses to determine predictors of intrahepatic recurrence-free survival and extrahepatic recurrence-free survival.

Variables	IHRFS			EHRFS		
	**Univariate**	**Multivariate**		**Univariate**	**Multivariate**	
	**P value**	**HR (95% CI)**	**P value**	**P value**	**HR (95% CI)**	**P value**
Gender (male vs female)	0.163			0.983		
Age (years)	0.209			0.965		
Primary tumor						
Location (left-sided vs right-sided)	0.228			0.968		
Stage (N1-2 vs N0)	0.320			0.470		
RAS/BRAF status (mutation vs wild-type)	0.560			0.003	2.85 (1.54-5.28)	0.001
Liver metastases						
Synchronous vs metachronous	0.951			0.275		
Distribution (bilobar vs unilobar)	0.756			0.939		
Number of lesions	0.028	1.02 (1.00-1.05)	0.028	0.533		
Maximum lesion size at diagnosis (mm)	0.180			0.697		
Extrahepatic metastases						
None	Ref			Ref	Ref	Ref
Lung	0.455			0.545	1.02 (0.42-2.45)	0.971
Others	0.963			0.039	3.43 (1.37-8.61)	0.009
CEA (ng/mL)	0.717			0.206		
Pre-treatment therapy						
First-line vs second-line	0.880			0.735		
Best response (SD vs PR)	0.460			0.567		

Abbreviations: IHRFS, intrahepatic recurrence-free survival; EHRFS, extrahepatic recurrence-free survival; HR, hazard ratio; CI, confidence interval; CEA, carcinoembryonic antigen; SD, stable disease; PR, partial response.

Table S4. Studies of hepatectomy alone or combined ablation and resection for CRLMs in the recent 5 years.

Study	Design	Period	Treatment	Sample Size	Endpoints
Joechle 2020 [1]	Retrospective	2006-2016	Hepatectomy	360	LF: 9.7%<br>Median PFS: 12m
Liu 2021 [2]	Retrospective	2016-2019	Hepatectomy	268	IHRFS: 1-year 65.7%<br>Median PFS: 12m
			CARe	67	Median IHRFS: 8m<br>Median PFS: 7m
Cheng 2022 [3]	Retrospective	1999-2020	Hepatectomy	90	Median PFS: 19m<br>Median OS: 45m
			CARe	8	
Canseco 2023 [4]	Multicenter	2004-2017	Hepatectomy	637	Median OS: 54m
			CARe	92	Median OS: 48m
Liu 2023 [5]	Retrospective	2000-2020	Hepatectomy	96	Median IHRFS: 11m<br>Median OS: 47m
			CARe	96	Median IHRFS: 10m<br>OS: 3-year 72.2%
Valenzuela 2023 [6]	Multicenter	2000-2018	Hepatectomy	906	Median OS: 46.8m
Pourfaraji 2024 [7]	Retrospective	2012-2022	Hepatectomy	248	Median OS: 46m
Mainali 2024 [8]	Multicenter	2000-2018	Hepatectomy	63	Median PFS: 22.7m<br>Median OS: 45.1m
			CARe	63	Median PFS: 14.2m<br>Median OS: 54.8m
Vadisetti 2024 [9]	Retrospective	2010-2022	Hepatectomy	258	LF: 7.0%<br>IHRFS: 3-year 54.4%<br>Median PFS: 13m<br>Median OS: 38m

Abbreviations: CARe, combined ablation and resection; LF, local failure; PFS, progression-free survival; IHRFS, intrahepatic recurrence-free survival; OS, overall survival.

References

1. Joechle, K.; Vreeland, T.J.; Vega, E.A.; Okuno, M.; Newhook, T.E.; Panettieri, E.; Chun, Y.S.; Tzeng, C.D.; Aloia, T.A.; Lee, J.E.; et al. Anatomic Resection Is Not Required for Colorectal Liver Metastases with RAS Mutation. *Journal of gastrointestinal surgery : official journal of the Society for Surgery of the Alimentary Tract* **2020**, *24*, 1033-1039, doi:10.1007/s11605-019-04299-6.
2. Liu, M.; Wang, K.; Wang, Y.; Bao, Q.; Wang, H.; Jin, K.; Liu, W.; Xing, B. Short- and long-term outcomes of hepatectomy combined with intraoperative radiofrequency ablation for patients with multiple primarily unresectable colorectal liver metastases: a propensity matching analysis. *HPB (Oxford)* **2021**, *23*, 1586-1594, doi:10.1016/j.hpb.2021.03.014.
3. Cheng, K.C.; Yip, A.S. Prognostic factors of survival and a new scoring system for liver resection of colorectal liver metastasis. *World J Hepatol* **2022**, *14*, 209-223, doi:10.4254/wjh.v14.i1.209.
4. Canseco, L.M.; Liu, Y.W.; Lu, C.C.; Lee, K.C.; Chen, H.H.; Hu, W.H.; Tsai, K.L.; Yang, Y.H.; Wang, C.C.; Hung, C.H. Survival Evidence of Local Control for Colorectal Cancer Liver Metastases by Hepatectomy and/or Radiofrequency Ablation. *Cancers* **2023**, *15*, doi:10.3390/cancers15184434.
5. Liu, M.; Wang, Y.; Wang, K.; Bao, Q.; Wang, H.; Jin, K.; Liu, W.; Yan, X.; Xing, B. Combined ablation and resection (CARe) for resectable colorectal cancer liver Metastases-A propensity score matching study. *European journal of surgical oncology : the journal of the European Society of Surgical Oncology and the British Association of Surgical Oncology* **2023**, *49*, 106931, doi:10.1016/j.ejso.2023.05.006.
6. Valenzuela, C.D.; Moaven, O.; Solsky, I.B.; Stauffer, J.A.; Del Piccolo, N.R.; Cheung, T.; Corvera, C.U.; Wisneski, A.D.; Cha, C.H.; Pourhabibi Zarandi, N.; et al. Conditional Survival After Hepatectomy for Colorectal Liver Metastasis: Results from the Colorectal Liver Operative Metastasis International Collaborative (COLOMIC). *Annals of surgical oncology* **2023**, *30*, 3413-3422, doi:10.1245/s10434-023-13189-w.
7. Pourfaraji, S.M.; Moghadam, M.N.; Moradi, A.M.; Shirmard, F.O.; Mohammadzadeh, N.; Jafarian, A. Long-term survival after hepatic resection for colorectal liver metastases: a single-center study in Iran. *BMC surgery* **2024**, *24*, 131, doi:10.1186/s12893-024-02420-4.
8. Mainali, B.B.; Valenzuela, C.D.; Moaven, O.; Stauffer, J.A.; Del Piccolo, N.R.; Cheung, T.; Corvera, C.U.; Wisneski, A.D.; Cha, C.H.; Zarandi, N.P.; et al. Resection versus resection with ablation: Analysis from the colorectal liver operative metastasis international collaborative. *Journal of surgical oncology* **2024**, *130*, 516-522, doi:10.1002/jso.27789.
9. Vadisetti, S.N.; Kazi, M.; Patkar, S.; Mundhada, R.; Desouza, A.; Saklani, A.; Goel, M. Patterns and Predictors of Recurrence After Curative Resection of Colorectal Liver Metastasis (CRLM). *Journal of gastrointestinal cancer* **2024**, *55*, 1559-1568, doi:10.1007/s12029-024-01105-8.

Table S5. Key clinical trials of first-line chemotherapy for initially unresectable CRLMs.

Study	Design	Sample Size	Regimen	Endpoints
CELIM [1]	Phase II	111	FOLFOX + Cet *vs*<br>FOLFIRI + Cet	R0 resection: 38% *vs* 30%<br>median PFS: 11.2m *vs* 10.5m<br>median OS: 35.8m *vs* 29.0m
TRIBE [2]	Phase III	508	FOLFOXIRI + Bev *vs*<br>FOLFIRI + Bev	median PFS: 12.3m *vs* 9.7m<br>median OS: 29.8m *vs* 25.8m
TRIBE-2 [3]	Phase III	679	FOLFOXIRI + Bev *vs*<br>mFOLFOX6 + Bev	median PFS: 12.0m *vs* 9.8m<br>median OS: 27.3m *vs* 22.5m
BECOME [4]	Phase II	241	mFOLFOX6 + Bev *vs*<br>mFOLFOX6	R0 resection: 22.3% *vs* 5.8%<br>median PFS: 9.5m *vs* 5.6m<br>median OS: 25.7m *vs* 20.5m
CAIRO5 [5]	Phase III	294	*right-sided or mutated*<br>FOLFOX/FOLFIRI + Bev<br>*vs* FOLFOXIRI + Bev	R0/R1 resection: 37% *vs* 51%<br>median PFS: 9.0m *vs* 10.6m<br>median OS: 23.6m vs 24.1m
		236	*left-sided and wild-type*<br>FOLFOX/FOLFIRI + Bev<br>*vs* + Pani	R0/R1 resection: 58% vs 58%<br>median PFS: 10.8m vs 10.4m<br>median OS: 40.4m vs 38.3m

Abbreviations: Cet, cetuximab; Bev, bevacizumab; Pani, panitumumab; PFS: progression-free survival; OS, overall survival.

References
1. Folprecht, G.; Gruenberger, T.; Bechstein, W.; Raab, H.R.; Weitz, J.; Lordick, F.; Hartmann, J.T.; Stoehlmacher-Williams, J.; Lang, H.; Trarbach, T.; et al. Survival of patients with initially unresectable colorectal liver metastases treated with FOLFOX/cetuximab or FOLFIRI/cetuximab in a multidisciplinary concept (CELIM study). *Annals of Oncology* **2014**, *25*, 1018-1025, doi:10.1093/annonc/mdu088.
2. Cremolini, C.; Loupakis, F.; Antoniotti, C.; Lupi, C.; Sensi, E.; Lonardi, S.; Mezi, S.; Tomasello, G.; Ronzoni, M.; Zaniboni, A.; et al. FOLFOXIRI plus bevacizumab versus FOLFIRI plus bevacizumab as first-line treatment of patients with metastatic colorectal cancer: updated overall survival and molecular subgroup analyses of the open-label, phase 3 TRIBE study. *Lancet Oncol* **2015**, *16*, 1306-1315, doi:10.1016/s1470-2045(15)00122-9.
3. Cremolini, C.; Antoniotti, C.; Rossini, D.; Lonardi, S.; Loupakis, F.; Pietrantonio, F.; Bordonaro, R.; Latiano, T.P.; Tamburini, E.; Santini, D.; et al. Upfront FOLFOXIRI plus bevacizumab and reintroduction after progression versus mFOLFOX6 plus bevacizumab followed by FOLFIRI plus bevacizumab in the treatment of patients with metastatic colorectal cancer (TRIBE2): a multicentre, open-label, phase 3, randomised, controlled trial. *Lancet Oncol* **2020**, *21*, 497-507, doi:10.1016/s1470-2045(19)30862-9.
4. Tang, W.; Ren, L.; Liu, T.; Ye, Q.; Wei, Y.; He, G.; Lin, Q.; Wang, X.; Wang, M.; Liang, F.; et al. Bevacizumab Plus mFOLFOX6 Versus mFOLFOX6 Alone as First-Line Treatment for RAS Mutant Unresectable Colorectal Liver-Limited Metastases: The BECOME Randomized Controlled Trial. *Journal of clinical oncology : official journal of the American Society of Clinical Oncology* **2020**, *38*, 3175-3184, doi:10.1200/jco.20.00174.
5. Bond, M.J.G.; Bolhuis, K.; Loosveld, O.J.L.; de Groot, J.W.B.; Droogendijk, H.; Helgason, H.H.; Hendriks, M.P.; Klaase, J.M.; Kazemier, G.; Liem, M.S.L.; et al. First-line systemic treatment strategies in patients with initially unresectable colorectal cancer liver metastases (CAIRO5): an open-label, multicentre, randomised, controlled, phase 3 study from the Dutch Colorectal Cancer Group. *Lancet Oncol* **2023**, *24*, 757-771, doi:10.1016/s1470-2045(23)00219-x.
